# Just-in-Time Adaptive Interventions for physical activity: a systematic review of their public health impact and methodological quality

**DOI:** 10.3389/fpubh.2026.1934024

**Published:** 2026-09-04

**Authors:** Shili Yang, Mingli Li, Yangfei Li, Yanhui Chang, Shuang Hu, Xinru Kou, Xiangrong Liu

**Affiliations:** College of Nursing, Changchun University of Chinese Medicine, Changchun, China

**Keywords:** digital health, Just-in-Time Adaptive Intervention, mobile health, physical activity, systematic review

## Abstract

**Background:**

Physical inactivity is a major public health challenge. Just-in-Time Adaptive Interventions (JITAIs) offer a promising digital approach to promote physical activity. However, the public health potential of JITAIs—their effectiveness and the quality of evidence supporting them—remains unclear. This review focuses on their potential for public health impact and the methodological quality of the evidence.

**Objective:**

To systematically review the effects of JITAIs on physical activity behaviors and to critically appraise the methodological quality of the evidence from a public health perspective.

**Methods:**

A systematic search was conducted in PubMed, Web of Science, Embase, Scopus, CINAHL, PsycINFO, and Cochrane CENTRAL from database inception to June 1, 2026. Interventional studies that employed JITAIs as the intervention strategy, measured physical activity as an outcome, and enrolled adults (≥18 years) were included. Two investigators independently screened studies and extracted data. Risk of bias for randomized controlled trials (RCTs) was assessed using the Cochrane RoB 2 tool. A narrative synthesis approach was used to present the findings.

**Results:**

A total of 37 studies were included, comprising 6,939 participants. Steps were the most frequently reported outcome, measured in 64.9% of the included studies. Supportive RCTs reported effect sizes ranging from +371 to +1,327 steps/day, although exploratory meta-analysis revealed substantial heterogeneity (*I*^2^ = 85%). For moderate-to-vigorous physical activity (MVPA), all RCTs that reported complete between-group data showed positive effects, with effect sizes ranging from +13 to +200 min/week. For sedentary time, two RCTs reported objectively measured data showing consistent direction of effect, although neither reached statistical significance.

**Conclusion:**

JITAIs show preliminary promise for promoting physical activity, but the evidence base is methodologically immature, characterized by substantial heterogeneity and inconsistent reporting. To realize their public health potential, future research must adopt standardized outcome reporting and prioritize head-to-head comparisons against static digital interventions to establish their true incremental value. These findings provide a clear roadmap for strengthening the evidence base needed to inform public health policy and practice.

**Systematic review registration:**

https://www.crd.york.ac.uk/PROSPERO/view/CRD420261432266, CRD420261432266.

## 1 Introduction

Physical inactivity is a leading modifiable risk factor for non-communicable diseases (NCDs) and a significant contributor to the global burden of disease (1–4). Despite robust evidence linking regular physical activity to reduced risks of cardiovascular disease, type 2 diabetes, certain cancers, and all-cause mortality, global progress in increasing population activity levels has stagnated (5). The World Health Organization (WHO) reports that approximately one in three adults and four in five adolescents fail to meet recommended physical activity guidelines, and the global target of a 15% relative reduction in inactivity by 2030 remains off track (6). In parallel, sedentary behavior—defined as prolonged waking time spent in seated or reclined postures with very low energy expenditure—has emerged as a distinct risk factor for chronic disease, independent of physical inactivity (7). Consequently, identifying effective, scalable, and cost-efficient strategies to promote physical activity across diverse populations has become a critical priority for public health policy and practice (8, 9).

In recent years, the widespread adoption of mobile health (mHealth) technologies—including smartphones, wearable sensors, and real-time monitoring systems—has created new opportunities for delivering personalized health interventions in real-world settings (10, 11). mHealth apps have been shown to be effective in increasing physical activity and MVPA levels and reducing sedentary behavior in older adults. Digital health interventions can effectively enhance daily activity levels in various populations (12). Among these innovations, Just-in-Time Adaptive Interventions (JITAIs) have emerged as a promising digital health strategy. Unlike traditional static digital interventions that deliver fixed content at predetermined times, JITAIs leverage real-time or near-real-time data from wearable devices and/or self-reports to dynamically assess an individual’s current state (e.g., physiological indicators, activity levels, environmental context) at predefined decision points and adapt the content, timing, or intensity of support accordingly (13, 14). The design and development of such personalized applications should be grounded in behavior change theories, end-user perceptions, and empirical data mining principles (15). This adaptive feature is theoretically expected to enhance the relevance and timeliness of interventions while minimizing user fatigue and intervention burnout—challenges commonly associated with ill-timed or generic health messaging.

Physical activity promotion is one of the most active application domains for JITAIs, with interventions targeting both the increase of moderate-to-vigorous physical activity (MVPA) and the reduction of sedentary behavior. Hardeman et al. conducted the first systematic review of JITAIs for physical activity, including 19 studies up to 2018, and concluded that the field was in its early stages, characterized by a weak evidence base and heterogeneous study quality (16). However, that review did not capture the rapidly expanding body of research published over the past 7 years. Since 2019, the number of JITAI-related studies has grown substantially, and new experimental designs—such as microrandomized trials and sequential multiple assignment randomized trials (SMART)—have been introduced to better evaluate these dynamic interventions (17–20). Recent systematic reviews have also examined the implementation of behavior change theories and techniques in mHealth JITAIs, as well as the effectiveness of mHealth-based interventions on physical activity and body composition (21). At the same time, a critical question remains unanswered: whether the added complexity and cost of JITAI adaptive features consistently translate into meaningful behavioral improvements beyond those achieved by simpler, lower-cost static digital interventions. For public health systems with finite resources, demonstrating this incremental value is essential before investing in the widespread deployment of adaptive digital interventions.

The present review advances the field in four distinct ways. First, unlike previous reviews that focused primarily on feasibility and descriptive characteristics of JITAIs (16), we specifically examine the incremental value of JITAIs relative to static digital interventions—a question of central importance for public health decision-making that has not been systematically addressed. Second, we incorporate emerging study types (MRTs and SMART designs) that were not represented in earlier reviews. Third, we provide an exploratory quantitative synthesis of step outcomes to assess the consistency of effects across studies. Fourth, we systematically appraise the methodological quality and reporting completeness of the included studies, with a focus on identifying gaps that may limit evidence accumulation, such as the consistency of outcome reporting and the availability of data suitable for synthesis. Therefore, this systematic review aimed to comprehensively synthesize the available evidence on the effects of JITAIs on physical activity behaviors in adult populations, with a specific focus on their potential public health impact and the methodological quality of the evidence base. Specifically, we sought to: (1) describe the effects of JITAIs on steps, MVPA, and sedentary time, stratified by study design; (2) critically appraise the methodological quality and reporting completeness of the included studies; and (3) examine the incremental effects of JITAIs relative to non-adaptive comparator conditions. By systematically mapping the current evidence landscape and identifying key methodological gaps, this review provides actionable insights for researchers, public health practitioners, and policymakers seeking to leverage digital technologies to support health behavior change in real-world settings.

## 2 Methods

### 2.1 Study design and registration

This systematic review was conducted and reported in accordance with the PRISMA 2020 guidelines. The study protocol was prospectively registered with PROSPERO (registration number: CRD420261432266).

### 2.2 Inclusion and exclusion criteria

The inclusion criteria were defined according to the PICOS framework:

- **Population**: Adults aged ≥18 years, regardless of health status.
- **Intervention**: Interventions meeting the operational definition of a JITAI—that is, driven by real-time or near-real-time individual state data, dynamically triggered at predefined decision points, and personalized in content and/or timing based on the individual’s current state (13).
- **Comparison**: No restrictions on comparator type, including static digital interventions, usual care, and others.
- **Outcome**: Physical activity and sedentary behavior indicators, including step counts, moderate-to-vigorous physical activity (MVPA), sedentary time, standing time, and others, assessed via objective measures or self-report.
- **Study design**: Interventional studies, including randomized controlled trials (RCTs), microrandomized trials, sequential multiple assignment randomized trials (SMART), single-arm pre-post studies, non-randomized controlled trials, and others.

Exclusion criteria were: (1) non-interventional studies (e.g., purely observational studies, cross-sectional surveys, qualitative studies); (2) studies not reporting physical activity behavior as a primary outcome; (3) secondary analyses or process evaluations of previously published original studies (e.g., mediation analyses, moderation analyses, subgroup analyses); and (4) conference abstracts, reviews, editorials, commentaries, study protocols, and other non-original research literature.

### 2.3 Search strategy

A systematic search was conducted in PubMed, Web of Science Core Collection, Embase, Scopus, CINAHL Ultimate (EBSCOhost), PsycINFO (EBSCOhost), and Cochrane CENTRAL, covering all records from database inception to June 1, 2026. Both MeSH terms and free-text terms were used. The PubMed search strategy is presented in Table 1. Supplementary searches were conducted in ACM Digital Library, DBLP, ClinicalTrials.gov, and ISRCTN using the core terms “Just-in-Time Adaptive Intervention” and “JITAI.” The full search strings for these supplementary databases are provided in Supplementary material S1. In addition, the reference lists of all included studies were manually searched to identify potentially eligible studies.

**Table 1:** PubMed search strategy.

Search number	Query
1	“Exercise”[Mesh]
2	Physical Activity [tw] OR Exercise [tw] OR Physical fit*[tw] OR Sport* participation[tw] OR Sport* activit* [tw] OR Active lifestyle[tw] OR Movement behavio*[tw] OR MVPA[tw] OR Moderate to vigorous physical activity [tw] OR Step count* [tw] OR Energy expenditur*[tw] OR Daily activit* [tw] OR Walk* [tw] OR Jog[tw] OR Run
3	(“Exercise”[Mesh]) OR (Physical Activity [tw] OR Exercise [tw] OR Physical fit*[tw] OR Sport* participation[tw] OR Sport* activit* [tw] OR Active lifestyle[tw] OR Movement behavio*[tw] OR MVPA[tw] OR Moderate to vigorous physical activity [tw] OR Step count* [tw] OR Energy expenditur*[tw] OR Daily activit* [tw] OR Walk* [tw] OR Jog[tw] OR Run[tw])
4	“just-in-time”[Title/Abstract] OR “JITAI*”[Title/Abstract] OR “ecological momentary intervention*”[Title/Abstract] OR “context-aware*”[Title/Abstract] OR “adaptive intervention”[Title/Abstract:~4] OR “adaptive interventions”[Title/Abstract:~4] OR “digital behavior change”[Title/Abstract] OR “digital behavior change”[Title/Abstract] OR “DBCI*”[Title/Abstract] OR “dynamic tailoring”[Title/Abstract:~4] OR “dynamically tailored”[Title/Abstract:~4] OR “real-time tailoring”[Title/Abstract:~4] OR “real-time therap*”[Title/Abstract] OR “tailored feedback”[Title/Abstract:~4] OR “adaptive feedback”[Title/Abstract:~4] OR “real-time feedback”[Title/Abstract:~4] OR “microrandomi*”[Title/Abstract] OR “micro-randomi*”[Title/Abstract]
5	((“Exercise”[Mesh]) OR (Physical Activity [tw] OR Exercise [tw] OR Physical fit*[tw] OR Sport* participation[tw] OR Sport* activit* [tw] OR Active lifestyle[tw] OR Movement behavio*[tw] OR MVPA[tw] OR Moderate to vigorous physical activity [tw] OR Step count* [tw] OR Energy expenditur*[tw] OR Daily activit* [tw] OR Walk* [tw] OR Jog[tw] OR Run)) AND (“just-in-time”[Title/Abstract] OR “JITAI*”[Title/Abstract] OR “ecological momentary intervention*”[Title/Abstract] OR “context-aware*”[Title/Abstract] OR “adaptive intervention”[Title/Abstract:~4] OR “adaptive interventions”[Title/Abstract:~4] OR “digital behavior change”[Title/Abstract] OR “digital behavior change”[Title/Abstract] OR “DBCI*”[Title/Abstract] OR “dynamic tailoring”[Title/Abstract:~4] OR “dynamically tailored”[Title/Abstract:~4] OR “real-time tailoring”[Title/Abstract:~4] OR “real-time therap*”[Title/Abstract] OR “tailored feedback”[Title/Abstract:~4] OR “adaptive feedback”[Title/Abstract:~4] OR “real-time feedback”[Title/Abstract:~4] OR “microrandomi*”[Title/Abstract] OR “micro-randomi*”[Title/Abstract])

### 2.4 Study selection and data extraction

Two investigators independently screened studies and extracted data based on the inclusion and exclusion criteria. Title and abstract screening, full-text screening, and data extraction were independently performed by two investigators, with disagreements resolved through discussion with a third investigator. Duplicates were removed using NoteExpress. Titles and abstracts were screened, and the full texts of potentially eligible studies were reviewed for final inclusion. Data were extracted using a standardized extraction form that was piloted prior to use. Extracted information included: study characteristics (first author, publication year, country); population characteristics (sample size, age, health status); intervention characteristics (JITAI design type, decision rules, data sources, intervention duration, level of human support); comparator conditions; and outcome measures and data.

### 2.5 Risk of bias assessment

The Cochrane Risk of Bias tool version 2.0 (RoB 2) was used to assess the risk of bias in included randomized controlled trials (22), covering five domains: randomization process, deviations from intended interventions, missing outcome data, measurement of the outcome, and selection of the reported result. Assessments were independently conducted by two investigators, with disagreements resolved through discussion with a third investigator. Microrandomized trials (MRTs) were not formally assessed using RoB 2 due to their within-participant repeated randomization design, which is methodologically distinct from traditional parallel-group RCTs, and the current lack of a standardized risk of bias tool for this design (23); instead, their potential sources of bias were described narratively. Non-randomized controlled trials were assessed using the ROBINS-I tool (24). Single-arm pre-post studies, feasibility studies, SMART designs, and multiple-baseline single-case designs were not formally assessed with risk of bias tools due to the absence of independent control groups or special design features; their methodological limitations were described narratively in the Discussion. Results from studies not formally assessed should be interpreted with caution. Risk of bias results for RCTs were presented as traffic light plots, generated using R version 4.5.0.

### 2.6 Data synthesis

Given the anticipated heterogeneity in study designs, comparator conditions, and outcome measures, a narrative synthesis approach was used to present the results. Findings were stratified by study design (RCTs, microrandomized trials, SMART designs, single-arm/feasibility studies). For RCTs that reported complete between-group data (mean differences and standard errors), exploratory random-effects meta-analyses were conducted to quantify heterogeneity (I^2^ statistic), with results presented for illustrative purposes only and not as definitive conclusions.

## 3 Results

### 3.1 Study selection

**Figure 1:**
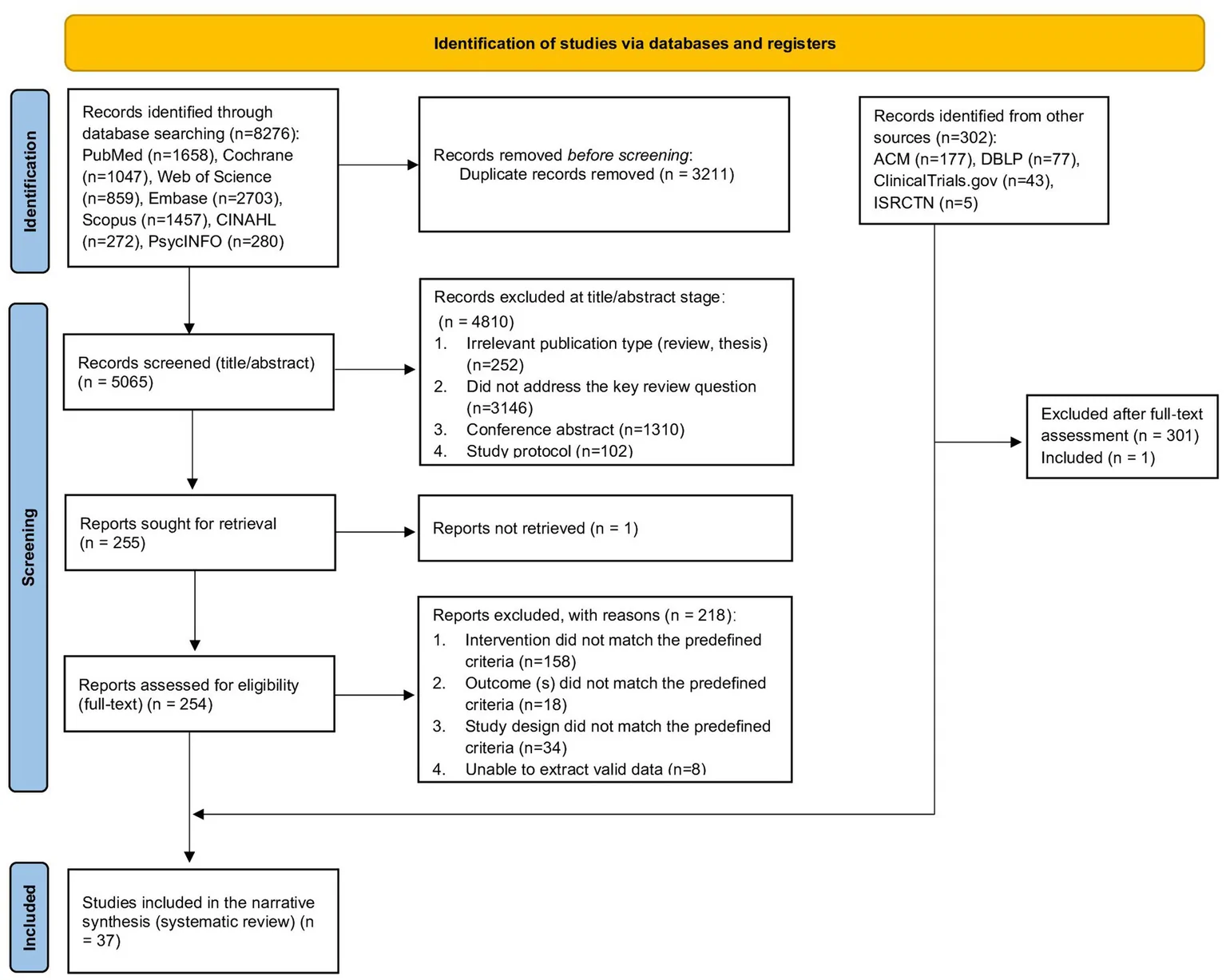
PRISMA 2020 flow diagram, including supplementary searches in ACM Digital Library, DBLP, ClinicalTrials.gov, and ISRCTN.

The initial search yielded 8,276 records. After deduplication, 5,065 records remained. Following title and abstract screening, 4,810 records were excluded, leaving 255 full-text articles for assessment. After full-text review, 218 articles were excluded, resulting in 36 studies meeting the inclusion criteria from the primary search. In addition, to ensure comprehensive coverage of interdisciplinary literature, we performed supplementary searches in ACM Digital Library, DBLP, ClinicalTrials.gov, and ISRCTN using the core terms “Just-in-Time Adaptive Intervention” and “JITAI.” These supplementary searches identified 302 additional records. After full-text assessment, 301 records were excluded because they were study protocols, methodological papers without empirical data, or did not target physical activity as a primary outcome. One additional study (25)—a concept validation study with no patient participants—was identified through the supplementary searches and retained to provide insight into an emerging JITAI direction (LLM-generated interventions), while being clearly distinguished from empirical studies in Table 2. The total number of included studies was 37 (17–20, 25–57). The study selection process is presented in the PRISMA flow diagram (Figure 1).

**Table 2:** Characteristics of included studies.

First author (year)	Country	Study design	Population	Sample size (I/C)	Comparator description	Key physical activity outcome findings
Sanal-Hayes (2026) (26)	UK	RCT	Adults with long COVID	250 (125/125)	Pace Me App (no Fitbit, no JITAI)	Steps, MVPA, sedentary time (objective Fitbit, between-group differences not reported)
Golbus (2023) (42)	USA	RCT	Patients with low-to-moderate risk CVD	220 (112/109)	Smartwatch + basic App	Steps (MD not significant at 3/6 months), 6-min walk distance
Thomas (2022) (45)	USA	RCT	Overweight/obese pregnant women	68 (33/35)	Usual prenatal care	MVPA (self-reported PPAQ, adjusted MD + 199.6 min/week)
Hedderson (2026) (27)	USA	Cluster RCT	Overweight/obese pregnant women	1,265 (677/588)	Usual prenatal care	MVPA (self-reported PPAQ, between-group differences not reported)
Huffman (2023) (35)	USA	Pilot RCT	Adults with ≥2 cardiac risk conditions	60 (31/29)	Omron pedometer + usual medical care	MVPA, steps (objective ActiGraph, Cohen’s *d* = 0.37 at 12 weeks)
Bronas (2024) (36)	USA	Pilot RCT	Community-dwelling Spanish-speaking Latino older adults	39 (20/19)	Physical activity guideline education video	Steps (+1,327 steps/day), MVPA (+82.7 min/week), sedentary time (−53.4 min/day)
Valle (2023) (43)	USA	RCT	Young adult cancer survivors	280 (140/140)	Self-help/digital tools (Fitbit + scale + website)	Steps (+371 steps/day), MVPA (+13.3 min/week), sedentary time (−3.0 min/day)
Wu (2026) (28)	China	RCT	Post-PCI patients with CHD	62 (31/31)	App-based standardized exercise video library + wearable ECG	Exercise duration, VO₂peak (non-behavioral PA)
Vos (2025) (30)	Netherlands	RCT	Community adults of low socioeconomic status	176 (87/89)	Step-counter only App	Steps (−202 steps/day, non-significant)
Seiterö (2025) (31)	Sweden	RCT	High school students (15–20 years)	756 (377/379)	Referral to Swedish national public health website (general health information only)	MVPA (+50.1 min/week, *p* = 0.05)
Lim (2011) (55)	South Korea	RCT	Older adults with type 2 diabetes	100 (50/50)	SMBG self-monitoring (no automatic upload/feedback)	Steps, MVPA (objective monitor, not reported separately)
Dorsch (2025) (33)	USA	RCT	Adults with self-reported hypertension	602 (298/304)	Fitbit + Omron BP monitor (no JITAI)	Steps (+489 steps/day, *p* = 0.04)
Wafi (2024) (39)	Saudi Arabia	RCT	Medical students	77 (37/40)	No intervention (education only in week 2)	Sedentary time (self-reported ACT24, within-group reduction of 1.82 h/day, no between-group difference)
Ikegaya (2025) (34)	Japan	Pilot RCT	Healthy university students	28 (14/14)	Unified intervention criteria (based on group mean thresholds)	Steps, MVPA, sedentary time (hourly means, interactions all non-significant)
King (2013) (57)	USA	Three-arm feasibility RCT	Community-dwelling older adults (≥45 years, physically inactive, sedentary, smartphone-naïve)	68 (Analytic 22/Social 23/Affect 23)	No no-intervention control; three apps compared (Analytic vs. Social vs. Affect)	MVPA (combined +188.6 min/week, *p* < 0.0001; between-group differences non-significant, *p* > 0.99); sedentary time (combined −29.1 min/day, *p* < 0.02; between-group differences non-significant, *p* > 0.34). Steps not reported.
Coppens (2024) (37)	Belgium	RCT	Healthy adults (18–65 years, not meeting PA recommendations)	44 (37/15)	Partially personalized algorithm (random selection)	Self-reported MVPA (group interaction non-significant, *p* > 0.05); recommended PA duration and intensity (significant group interaction, *p* < 0.001)
Sporrel (2022) (48)	Netherlands	RCT	Adults (18–55 years, living near parks)	20 (11/19)	Basic PAUL (reminders sent at random times, non-JITAI adaptive)	Objective MVPA (running/walking): between-group difference non-significant (*Z* = −1.433, *p* = 0.08). Within-group increases in intrinsic motivation for running/walking (*p* < 0.001) and strength training (*p* = 0.04), and perceived capability for strength training (*p* = 0.01); between-group differences all non-significant.
Oba (2025) (32)	Japan	RCT	Community adults (18–64 years, no regular exercise)	40 (20/20)	EMA only (no tailored messages)	HRR ≥ 40% (+16.03 min/day, *p* = 0.02, *d* = 0.35); steps (*p* = 0.24); self-reported PA (*p* = 0.44)
Phillips (2026) (17)	Canada	MRT	3–4 year old children and their full-time parents	25 dyads	Same EMA, no activity suggestions	Children and parents ST, LPA, MVPA (ACE all non-significant)
Klasnja (2019) (52)	USA	MRT	Healthy sedentary adults	37	No suggestion (randomized at same decision point)	Steps (30 min window: walking suggestion +59 steps, *p* = 0.02)
Klasnja (2020) (49)	USA	MRT	Post-bariatric surgery patients	23	Pre-post design; no suggestion within MRT	Steps (pre-post +1,866 steps/day; MRT proximal effect non-significant)
Wang (2023) (47)	USA	Cluster MRT	Medical interns	1,779	Non-competition weeks (dashboard viewable)	Steps (+106 steps/day during competition weeks, *p* = 0.03)
Höhener (2026) (18)	Switzerland	MRT	Romantic couples (cohabiting)	76	Control phase (no dyadic intervention)/no JITAI days	MVPA (+5.88 min/day during intervention phase; JITAI for self +11.17)
Golbus (2024) (40)	USA	MRT	Patients with CVD (cardiac rehabilitation)	108	No text messages (randomized at same decision point)	Steps (60 min window: Apple +10% during initiation, Fitbit +17% during initiation)
Golbus (2026) (29)	USA	MRT	Adults with self-reported hypertension	287	No notification (randomized at same decision point)	Steps (60 min window: main effect non-significant; low activity subgroup +4%)
Simoes (2026) (19)	Brazil	SMART	Physically inactive adults	53 (17/19/17)	Educational information (video + manual)	Steps, sedentary time, MVPA (objective ActiGraph)
Whiston (2025) (20)	Ireland	SMART	Community-dwelling stroke survivors	50 (28/22)	No traditional control (four EAIs compared)	Steps (marginal means no significant between-group differences)
Fiedler (2022) (41)	Germany	Single-arm pre-post	General healthy families (adults + children)	80	Within-subject control (non-responders)	Steps (60 min window: engaged +113 steps, *p* = 0.022); MET (+5.52, *p* = 0.014)
Mair (2022) (44)	UK	Single-arm pre-post	Community-dwelling older adults	31	None (single-arm design)	Feasibility outcomes primarily; PA data not fully reported
Hiremath (2019) (53)	USA	Single-arm pre-post	Individuals with spinal cord injury	20	None (single-arm design)	Moderate PA (within-group change non-significant)
Tabak (2014) (56)	Netherlands	Single-arm pre-post	Patients with COPD	15	None (single-arm design)	Activity counts (adjusted +13%, *p* = 0.008)
Gabrys (2016) (54)	Germany	Single-arm pre-post	Breast/colon cancer patients	19	Cross-sectional healthy control comparison	Total PA (cpm + 21%); MVPA (+9%)
Direito (2019) (51)	New Zealand	Single-arm pre-post	Adults not meeting PA recommendations	69	None (single-arm design)	MVPA (+7.7 min/day, *p* = 0.11); LPA (+2.2, *p* = 0.002)
Cushing (2021) (50)	USA	Non-randomized controlled	Community-dwelling adolescents	40 (20/20)	Attention control (wore accelerometer, no intervention)	MVPA (+20.8 min/day, *p* = 0.01); sedentary time (−82 min/day)
Ismail (2022) (46)	Qatar	Non-randomized controlled	Sedentary office workers	58 (29/29)	MotiFit Lite (static messages)	Sedentary behavior break rate (53.2% vs. 19.9%, *p* < 0.001)
Hendrickx (2024) (38)	Netherlands	Randomized multiple-baseline single-case	Community-dwelling stroke survivors	14 (7/7)	No participatory support group	Sedentary time (−1.3 h/day, *p* = 0.01); LPA; MVPA
Haag (2025) (25)	Austria/Germany	Concept validation study	Three personas representing patients with CVD of varying severity (low to high risk)	Concept validation study (no patient participants)	GPT-4-generated JITAIs vs. HCP-generated JITAIs vs. LayP-generated JITAIs (three-group comparison)	Not applicable (no steps/MVPA/sedentary time reported). GPT-4 significantly outperformed both HCPs and laypersons across all quality dimensions (appropriateness, engagement, effectiveness, and professionalism; all *p* < 0.001).

### 3.2 Characteristics of included studies

The 37 included studies comprised a total sample of 6,939 participants. The distribution of study designs was as follows: 18 RCTs (48.6%), 7 microrandomized trials (18.9%), 2 SMART designs (5.4%), 2 non-randomized controlled trials (5.4%), 6 single-arm/pre-post/feasibility studies (16.2%), and 1 multiple-baseline single-case design (2.7%), with 1 concept validation study (2.7%). The studies covered a wide range of health statuses and disease populations, including healthy adults, older adults, patients with cardiovascular disease, patients with type 2 diabetes, adults with overweight/obesity, pregnant and postpartum women, stroke survivors, individuals with spinal cord injury, patients with COPD, and cancer survivors. Studies were predominantly conducted in the United States (41.7%), Europe (33.3%), and Asia (16.7%). Detailed characteristics of the included studies are presented in Table 2.

### 3.3 JITAI design characteristics

The 37 studies exhibited substantial heterogeneity in JITAI design features, with detailed distributions presented in Table 3. Regarding decision rule types, threshold/If-Then rules were the most common (35.1%, 13 studies), followed by context-adaptive rules (29.7%, 11 studies) and adaptive goal/stepwise rules (16.2%, 6 studies); all other rule types each accounted for less than 6%. When categorized by algorithmic complexity, 19 studies (51.4%) used simple rule-based decision rules (threshold/if-then or adaptive goal/stepwise), while 17 studies (45.9%) employed context-aware or AI-driven algorithms (including context-adaptive, CDSS, reinforcement learning, LLM-based, and combined MRT designs), and 1 study (2.7%) could not be clearly classified. This distribution reflects the field’s ongoing evolution from simple rule-based systems toward more complex, data-driven approaches.

**Table 3:** Classification of JITAI design characteristics.

Design feature	Category	Number of studies (*n*)	Proportion (%)
Decision rule type			
Simple (rule-based)	Threshold/if-then rules	13	35.1
	Adaptive goal/stepwise rules	6	16.2
Complex (context-aware/AI-Driven)	Context-adaptive rules	11	29.7
	CDSS rule engine	2	5.4
	Reinforcement learning/adaptive ML	2	5.4
	Adaptive goal/stepwise + MRT	1	2.7
	LLM/Generative AI	1	2.7
Unclear	Feedback algorithm/user rating-driven	1	2.7
Data source type	Wearable devices (accelerometer)	20	54.1
	Smartphone-embedded sensors	5	13.5
	Multi-source fusion (wearable + EMA + GPS, etc.)	5	13.5
	Self-report/EMA	2	5.4
	Wearable + medical devices (ECG/glucometer)	2	5.4
	Scenario-based/simulated contexts	1	2.7
	Not explicitly reported	2	5.4
Human support	Fully automated	23	62.2
	Mixed model (automation + human coaching)	12	32.4
	Human-centric support	2	5.4
Intervention duration	≤4 weeks	8	21.6
	5–12 weeks	12	32.4
	13–24 weeks	7	18.9
	≥25 weeks	8	21.6
	Not explicitly reported	1	2.7
	Not applicable (concept validation study)	1	2.7
Message push frequency	Fixed-timing (daily/weekly scheduled)	13	35.1
	Event-triggered	9	24.3
	Mixed timing + event-triggered	8	21.6
	Random/adaptive frequency	3	8.1
	Not explicitly reported	3	8.1
	Not applicable (concept validation study)	1	2.7

*n* = 37. Proportion = number of studies/37 × 100%. Decision rule types are grouped by algorithmic complexity: ‘Simple’ refers to static rule-based thresholds without real-time adaptation; ‘Complex’ refers to algorithms that incorporate contextual data, machine learning, or real-time biosignal processing; ‘Unclear’ indicates insufficient information to classify. Haag et al. (25) is a concept validation study and was not included in the calculation of intervention duration or message push frequency.

Regarding data sources, wearable devices (accelerometers) were the predominant source (55.6%, 20 studies), followed by smartphone-embedded sensors and multi-source fusion (each 13.9%, 5 studies). Regarding human support, fully automated interventions accounted for the majority (61.1%, 22 studies), followed by mixed models (automation + periodic human coaching; 33.3%, 12 studies) and human-centric support (5.6%, 2 studies). Regarding intervention duration, 5–12 weeks was the most common (33.3%, 12 studies), followed by ≤4 weeks and ≥25 weeks (each 22.2%, 8 studies), and 13–24 weeks (19.4%, 7 studies). Regarding message push frequency, fixed-timing (daily/weekly scheduled) was the most common (36.1%, 13 studies), followed by event-triggered (25.0%, 9 studies), mixed timing and event-triggered (22.2%, 8 studies), and random/adaptive frequency or unreported (each 8.3%, 3 studies). The substantial heterogeneity across these design dimensions suggests that the field has not yet reached a standardized consensus on core JITAI design elements, limiting the comparability and cumulative evidence across studies.

### 3.4 Risk of bias assessment

**Figure 2:**
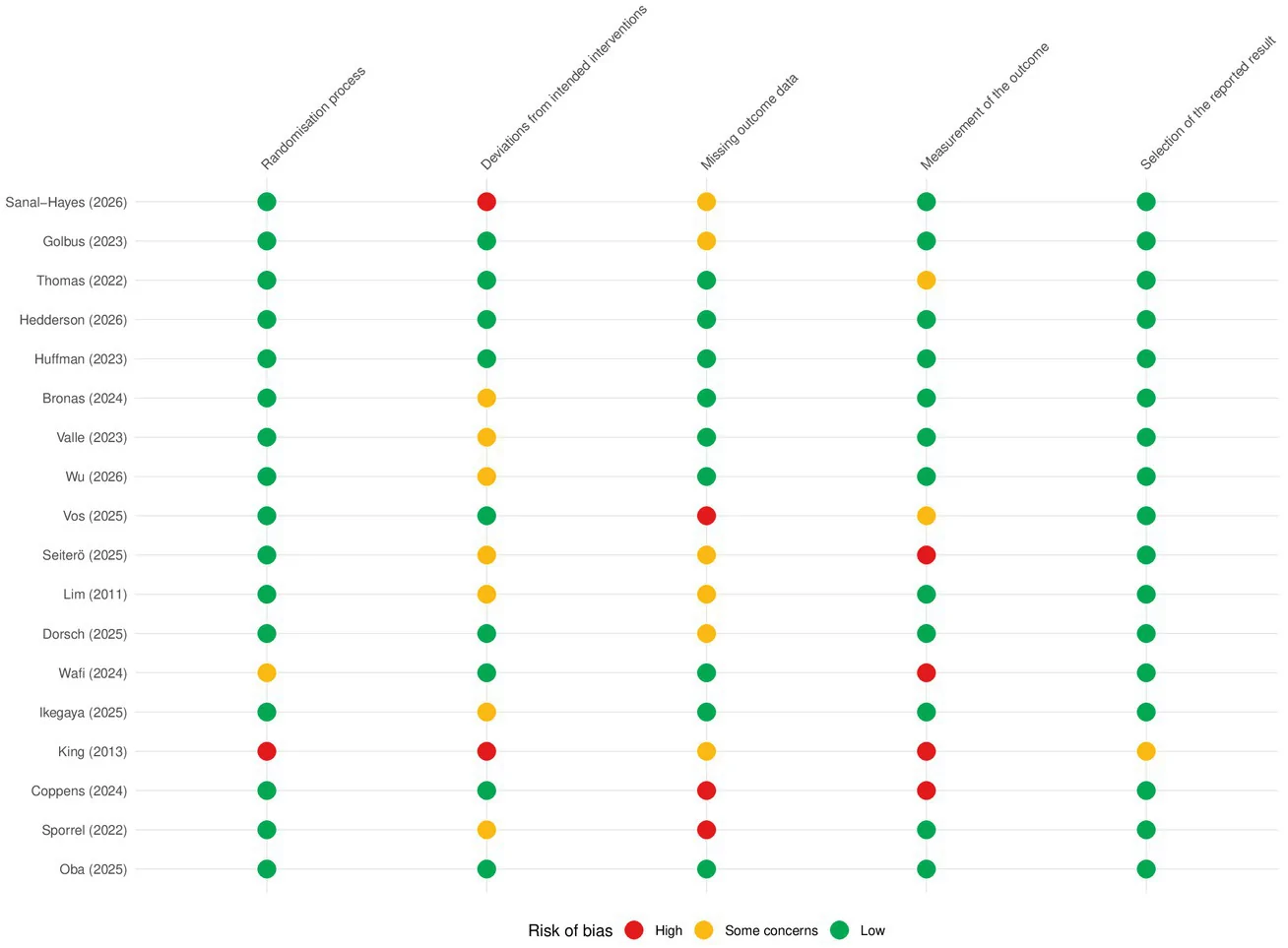
Risk of bias assessment results for included randomized controlled trials.

Risk of bias was assessed using the Cochrane RoB 2 tool for the 18 RCTs, with the distribution of risk levels across the five domains presented in Figure 2. Overall, 3 RCTs were judged to be at low risk of bias, 8 at moderate risk, and 7 at high risk. The main sources of high risk were missing outcome data and measurement of the outcome. Deviations from intended interventions was the most common source of bias in moderate-risk studies (7 studies). Two non-randomized controlled trials assessed using ROBINS-I were both judged to be at moderate risk of bias. For the six included MRTs, risk of bias was assessed narratively. Although MRTs reduce between-person confounding through within-participant randomization, other sources of bias remain, including potential carryover effects between decision points, variable participant adherence to prompts, and missing data at the decision-point level. None of the included MRTs reported formal assessments of these risks. Therefore, while MRTs provide valuable evidence on proximal intervention effects, their findings should be interpreted cautiously, and the development of MRT-specific quality assessment tools is needed for future syntheses.

### 3.5 Physical activity outcomes

#### 3.5.1 Step counts

Steps were the most frequently reported physical activity outcome, reported in approximately 64.9% of studies (24/37). Four RCTs provided complete between-group data suitable for quantitative synthesis, of which three supported the step-increasing effect of JITAIs (33, 36, 43) and one did not observe a positive effect (30), with effect sizes ranging from −202 to +1,327 steps/day. Exploratory meta-analysis yielded an overall effect of +534 steps/day (95% CI: −79 to 1,147), with substantial heterogeneity (*I*^2^ = 85%). The remaining RCTs could not be included due to incomplete reporting. Four MRTs reported positive proximal effects (18, 40, 47, 52)—defined as immediate behavioral changes measured within 30–60 min following a JITAI prompt (e.g., steps taken in the 30-min window after a walking suggestion)—while three did not observe any change in outcomes between groups (17, 29, 49). The inconsistency in effect direction may be related to differences in target populations (larger effects in chronic disease populations compared with general healthy populations), comparator conditions (smaller incremental effects when non-adaptive interventions were used as comparators), and intervention duration. Overall, most studies showed that JITAIs were favorable for increasing step counts; however, the large variability in effect sizes and high heterogeneity (*I*^2^ = 85%) limit the cumulative interpretation of the evidence.

#### 3.5.2 Moderate-to-vigorous physical activity (MVPA)

Approximately 40.5% of studies (15/37) reported MVPA data. Three RCTs provided complete between-group data, all showing effects favorable to JITAIs (31, 43, 45), with effect sizes ranging from +13 to +200 min/week. No RCTs reporting negative effects were identified. One non-randomized controlled trial (50) reported an increase of +20.8 min/day in MVPA (*p* = 0.01, *d* = 0.83). Among two MRTs, Höhener et al. (18) reported that JITAI-triggered messages increased MVPA by 11.17 min/day for the recipient (*p* < 0.001), while Phillips et al. (17) did not observe any change in outcomes between groups. Overall, the available evidence suggests that JITAIs may have positive short-term effects on MVPA; however, the precision of effect estimates is limited, and most findings are based on self-report measures, warranting further high-quality studies for confirmation.

#### 3.5.3 Sedentary time

Approximately 32.4% of studies (12/37) reported sedentary time data. Among the RCTs reporting objectively measured sedentary time, none demonstrated statistically significant reductions between groups (*p*-values ranging from 0.07 to 0.24) (17, 36, 39). One non-randomized study reported that the sedentary behavior break rate was significantly higher in the context-aware message group compared with the static message group (53.2% vs. 19.9%, *p* < 0.001) (46), and one multiple-baseline single-case design reported a within-participant reduction of 1.3 h/day in sedentary time (*p* = 0.01) (38).

### 3.6 Potential factors influencing JITAI effects

Based on preliminary observations from the available data, this review summarizes potential factors that may influence intervention effects from three aspects: comparator conditions, population characteristics, and JITAI design features. Regarding comparator conditions, the intensity of comparator settings varied considerably. Studies using usual care or no intervention as comparators (33, 36) showed relatively larger effect sizes, while studies using static digital interventions (e.g., step-counter only Apps) as comparators (30) showed smaller or even negative incremental effects. This pattern suggests that JITAIs may have some net benefit relative to minimal-intensity comparators; however, their incremental value over static digital interventions requires further head-to-head comparisons. Regarding population characteristics, effect sizes appeared to be larger in chronic disease or post-surgical populations compared with general healthy populations. Bronas et al. (36) and Dorsch et al. (33) reported step increases of +1,327 and +489 steps/day, respectively, in cardiovascular disease-related populations, whereas Vos et al. (30) did not observe any change in outcomes between groups in a general community adult population. This trend suggests that the effects of JITAIs may be influenced by participants’ baseline activity levels or health management motivation; however, current evidence is insufficient to support definitive subgroup difference inferences. Regarding JITAI design features, the influence of decision rule types, data source types, and levels of human support on intervention effects remains difficult to assess, primarily due to limited studies within each dimension and multiple confounders across studies, precluding meaningful between-group comparisons.

## 4 Discussion

### 4.1 Main findings

This systematic review synthesized evidence from 37 studies involving 6,939 participants to evaluate the effects of JITAIs on physical activity and to assess the methodological quality of the evidence base. Overall, JITAIs demonstrated preliminary positive trends—supportive RCTs showed increases in daily steps and weekly moderate-to-vigorous physical activity—with limited and inconclusive evidence for reducing sedentary time. However, the magnitude of these effects varied considerably across studies, and the overall direction of effect, while promising, was not uniform. Critically, the evidence base is methodologically immature. Fewer than one in four RCTs reported complete between-group step data suitable for quantitative synthesis, and most studies were at moderate or high risk of bias.

### 4.2 Evidence synthesis and research perspectives

Hardeman et al. published the first systematic review of JITAIs for physical activity in 2019, including 14 studies, and concluded that the field was in its early stages with insufficient evidence quality (16). The present review substantially extends this evidence base to 37 studies, capturing the rapid growth of research conducted since 2019. Unlike Hardeman et al., this review incorporates emerging study types—microrandomized trials and SMART designs—and focuses specifically on the incremental value of JITAIs relative to static digital interventions, a question of critical importance for public health decision-making. Additionally, we conducted an exploratory quantitative synthesis for step outcomes (*I*^2^ = 85%), providing empirical evidence that the field is not yet ready for definitive conclusions. Recent scoping reviews have similarly highlighted the need for more rigorous evaluation of behavior change theories and techniques in JITAI design (21).

Despite a near-doubling of the number of studies over the past 7 years, methodological standardization in the field remains limited. The persistent heterogeneity in study designs, comparator conditions, and outcome reporting underscores the need for concerted efforts to strengthen the evidence base before JITAIs can be confidently recommended for widespread implementation in real-world settings. Future research should prioritize: (1) head-to-head comparisons against static digital interventions to isolate the true incremental value of adaptive features; (2) standardized core outcome reporting to facilitate evidence synthesis; and (3) integration of proximal and distal outcomes in MRTs to establish whether immediate effects accumulate into sustained behavior change.

### 4.3 Implications for public health research and practice

The findings of this review have several implications for advancing the design and evaluation of JITAIs as individual-level interventions. The preliminary positive trends across multiple outcomes suggest that JITAIs hold promise as a public health tool, yet the substantial heterogeneity and lack of standardization mean they are not yet ready for widespread implementation. Public health practitioners should approach JITAIs with cautious optimism, recognizing their potential while awaiting more definitive evidence (25). From an implementation perspective, while JITAIs are delivered as individual-level interventions, scaling them to reach larger populations would require overcoming substantial barriers. Given the marginal or unclear incremental benefits observed over static digital interventions, it is currently unclear whether the additional complexity and cost of JITAIs can be justified over cheaper, readily implementable static apps, particularly given that JITAIs are delivered at the individual level and require substantial per-user resources. The additional costs associated with continuous sensor monitoring, real-time data processing, and algorithm maintenance may not be offset by the modest behavioral gains currently reported. Future implementation research must therefore embed formal cost-effectiveness analyses and budget-impact evaluations to determine whether the technological sophistication of JITAIs offers genuine value-for-money in real-world public health settings.

A critical unresolved question concerns the incremental value of JITAIs over simpler, less costly static digital interventions—studies using static comparators showed smaller or even negative effects. For public health systems with finite resources, establishing this added value is essential before investing in adaptive systems. Future RCTs must therefore employ static digital interventions as comparators to isolate the true incremental value of adaptive features. Moreover, the feasibility of deploying JITAIs on a larger scale warrants careful consideration. Over half of the interventions in this review relied on accelerometer-based wearables, whose widespread use faces significant barriers including cost, digital literacy, device adherence, and data privacy. Unlike static digital interventions delivered via simple text messaging, JITAIs require continuous sensor monitoring and real-time processing—all of which add complexity and cost. The limited evidence of incremental benefit over static alternatives suggests that scaling JITAIs for widespread use may not yet be a cost-effective strategy, particularly given the substantial per-user resources required.

The poor reporting quality identified in this review—only one in five RCTs provided complete between-group data—underscores an urgent need for standardized core outcome reporting. Specifically, future studies should consistently report group-specific means and standard deviations, between-group mean differences with 95% confidence intervals, and complete information on study design and comparator conditions. MRTs offer unique insights into proximal effects, yet their contribution is limited by inconsistent reporting of distal outcomes. We encourage MRT studies to report distal outcomes alongside proximal effects to clarify whether immediate effects accumulate into sustained long-term behavior change. Finally, the observed differences in effect sizes across populations—larger effects in chronic disease or post-surgical populations compared with general healthy populations—suggest that JITAIs may be most effective when targeted at individuals with specific health needs. This preliminary finding could inform more efficient resource allocation, although it requires confirmation through well-designed subgroup analyses. Population-level evidence further suggests that physical activity preferences vary across demographic groups (58), which may influence JITAI responsiveness and should be considered in future intervention design.

### 4.4 Limitations

This systematic review has several limitations that should be considered when interpreting the findings. A key limitation is that a formal meta-analysis was not conducted due to the substantial heterogeneity in study designs, comparator conditions, and outcome reporting formats, as well as the limited number of studies available for quantitative synthesis. Consequently, a pooled effect size estimate for JITAIs could not be provided. The high heterogeneity observed for step outcomes (*I*^2^ = 85%) could not be explored through subgroup analysis or meta-regression due to the limited number of studies, constraining our understanding of factors that may moderate JITAI effectiveness.

The certainty of the evidence is also constrained by the risk of bias profile of the included studies. The majority of RCTs (15 out of 18, 83%) were judged to be at moderate or high risk of bias—primarily driven by missing outcome data and outcome measurement biases—meaning that the observed effect estimates may be systematically overestimated or underestimated. Single-arm and feasibility studies lacked independent control groups, and MRTs currently lack standardized quality assessment tools; therefore, their risk of bias was assessed narratively in this review (as presented in Results 3.4). However, the absence of validated tools for these designs limits cross-study comparisons and underscores the need for future methodological development.

Language bias may also be present, as only English-language publications were included, and formal publication bias testing was not performed due to the insufficient number of studies available for meta-analysis (only 4).

Several factors may explain why reductions in sedentary time did not reach statistical significance in the RCTs. First, only two RCTs provided objectively measured sedentary time data, which substantially limited statistical power to detect small-to-moderate effects. Second, measuring sedentary behavior accurately requires specialized hip-worn accelerometers (e.g., activPAL), which are less commonly used than wrist-worn devices in physical activity studies, resulting in fewer studies with valid sedentary time data. Third, most JITAIs in this review were primarily designed to increase physical activity (steps and MVPA) rather than specifically target sedentary behavior as the primary outcome. These findings suggest that future JITAIs specifically designed to reduce sedentary time, with adequate sample sizes and objective measurement, are needed to determine whether JITAI can effectively reduce prolonged sitting.

We also acknowledge that the inclusion of Haag et al. (25)—a concept validation study with no patient participants—represents a departure from our PICOS criteria. This study was identified through our supplementary searches in computer science databases (ACM and DBLP) and was retained because it represents an emerging methodological direction in the JITAI field (i.e., the use of generative AI to issue tailored interventions). However, we have clearly labeled it as a concept validation study in Table 2, excluded it from the participant count, and urge readers to interpret its findings with appropriate caution. Additionally, while we included studies reporting sedentary behavior and feasibility outcomes to provide a comprehensive overview of JITAI applications, we acknowledge that these outcomes do not strictly represent physical activity promotion in the traditional sense [e.g., (39, 44, 46)]. This decision may have introduced heterogeneity in the narrative synthesis; however, we opted for inclusivity given the exploratory nature of this emerging field and have clearly distinguished these outcomes from primary physical activity measures in our presentation.

Despite these limitations, this review provides the most comprehensive and up-to-date synthesis of the evidence on JITAIs for physical activity, identifies critical gaps in the current evidence base, and offers clear recommendations for strengthening future research.

## Data Availability

The original contributions presented in the study are included in the article/Supplementary material, further inquiries can be directed to the corresponding author.

## References

[1] Yang L Zhang Z Zhang J Miao J Zhang H Du Y . Stage-specific risk factors of cardiometabolic multimorbidity: a systematic review and meta-analysis from incidence to mortality. Ageing Res Rev. (2026) 114:102991. doi: 10.1016/j.arr.2025.102991, 41390099

[2] Franssen WMA Nieste I Verboven K Eijnde BO. Sedentary behaviour and cardiometabolic health: integrating the potential underlying molecular health aspects. Metabolism. (2025) 170:156320. doi: 10.1016/j.metabol.2025.156320 40483777

[3] Sabag A Ahmadi MN Francois ME Postnova S Cistulli PA Fontana L . Timing of moderate to vigorous physical activity, mortality, cardiovascular disease, and microvascular disease in adults with obesity. Diabetes Care. (2024) 47:890–7. doi: 10.2337/dc23-2448, 38592034 PMC11043226

[4] Paluch AE Bajpai S Bassett DR Carnethon MR Ekelund U Evenson KR . Daily steps and all-cause mortality: a meta-analysis of 15 international cohorts. Lancet Public Health. (2022) 7:e219–28. doi: 10.1016/S2468-2667(21)00302-9, 35247352 PMC9289978

[5] World Health Organization. Physical activity. Geneva: World Health Organization.(2024). Available from: https://www.who.int/news-room/fact-sheets/detail/physical-activity (Accessed August 30, 2026).

[6] World Health Organization. WHO highlights high cost of physical inactivity in first-ever global report. Geneva: World Health Organization.(2022). Available from: https://www.who.int/news/item/19-10-2022-who-highlights-high-cost-of-physical-inactivity-in-first-ever-global-report (Accessed August 30, 2026).

[7] Guo L Wang C. The effect of exercise on cardiovascular disease risk factors in sedentary population: a systematic review and meta-analysis. Front Public Health. (2025) 13:1470947. doi: 10.3389/fpubh.2025.1470947, 40443938 PMC12119565

[8] Muhlis ANA Mahrouseh N Andrade CAS Chen-Xu J Eikemo TA Balaj M . Potential impact of increasing physical activity on NCD mortality in the EU: pathways to SDG 3.4.1 by 2030. BMJ Glob Health. (2026) 11:e022998. doi: 10.1136/bmjgh-2025-022998, 42097705 PMC13157740

[9] Mi MY Perry AS Krishnan V Nayor M. Epidemiology and cardiovascular benefits of physical activity and exercise. Circ Res. (2025) 137:120–38. doi: 10.1161/CIRCRESAHA.125.325526, 40608856 PMC12233137

[10] Laffi A Persiani M Piras A Meoni A Raffi M. Effectiveness of wearable technologies in supporting physical activity and metabolic health in adults with type 2 diabetes: a systematic-narrative hybrid review. Healthcare (Basel, Switzerland). (2025) 13:2422. doi: 10.3390/healthcare13192422, 41095508 PMC12524254

[11] Daniels K Quadflieg K Robijns J De Vry J Van Alphen H Van Beers R . From steps to context: optimizing digital phenotyping for physical activity monitoring in older adults by integrating wearable data and ecological momentary assessment. Sensors (Basel). (2025) 25:858. doi: 10.3390/s25030858, 39943497 PMC11820068

[12] Wang G Xiang R Yang X Tan L. Digital technology empowers exercise health management in older adults: a systematic review and meta-analysis of the effects of mHealth-based interventions on physical activity and body composition in older adults. Front Public Health. (2025) 13:1661028. doi: 10.3389/fpubh.2025.1661028, 41103467 PMC12521191

[13] Nahum-Shani I Murphy SA. Just-in-Time Adaptive Interventions: where are we now and what is next? Annu Rev Psychol. (2026) 77:679–703. doi: 10.1146/annurev-psych-121024-044244, 40939059 PMC13217361

[14] Nahum-Shani I Smith SN Spring BJ Collins LM Witkiewitz K Tewari A . Just-in-Time Adaptive Interventions (JITAIs) in mobile health: key components and design principles for ongoing health behavior support. Ann Behav Med. (2018) 52:446–62. doi: 10.1007/s12160-016-9830-8, 27663578 PMC5364076

[15] Sporrel K De Boer R Wang S Nibbeling N Simons M Deutekom M . The design and development of a personalized leisure time physical activity application based on behavior change theories, end-user perceptions, and principles from empirical data mining. Front Public Health. (2021) 8:528472. doi: 10.3389/fpubh.2020.528472, 33604321 PMC7884923

[16] Hardeman W Houghton J Lane K Jones A Naughton F. A systematic review of Just-in-Time Adaptive Interventions (JITAIs) to promote physical activity. Int J Behav Nutr Phys Act. (2019) 16:31. doi: 10.1186/s12966-019-0792-7, 30943983 PMC6448257

[17] Phillips SM Bourke M Inniss BV Ahluwalia M Tucker P. A pilot effectiveness study of a just-in-time micro-randomized controlled trial on the physical activity and sedentary time of young children and their parents: the active family m-health intervention. PLoS One. (2026) 21:e0340687. doi: 10.1371/journal.pone.0340687 PMC12810825 41544066

[18] Höhener PS Tobias R Allen JM Küng P Scholz U. Effectiveness of smartphone-based dyadic interventions to increase physical activity in romantic couples: microrandomized trial. JMIR Mhealth Uhealth. (2026) 14:e67136. doi: 10.2196/67136 PMC12892032 41592319

[19] Simoes MDSM Proença NL Lauria VT Do Nascimento MB Padovani RDC Dourado VZ. Effects of using a smartphone app combined with behavior change techniques on the level of physical activity among adults and older adults: sequential multiple assignment randomized trial. J Med Internet Res. (2026) 28:e73388. doi: 10.2196/73388 PMC12857902 41616115

[20] Whiston A Carr E Cardy N Rocliffe P O'Reilly S. M. Carter D . The Adaptive Physical Activity Study in Stroke (TAPAS); a Feasibility Sequential Multiple Assignment Randomized Trial. Weinheim, Germany: Wiley-VCH. e13316.(2025). 10.1002/advs.202513316 PMC12767112 41124672

[21] Cotie P Willms A Liu S. Implementation of behavior change theories and techniques for physical activity Just-in-Time Adaptive Interventions: a scoping review. Int J Environ Res Public Health. (2025) 22:1133. doi: 10.3390/ijerph22071133, 40724199 PMC12295828

[22] Sterne JAC Savović J Page MJ Elbers RG Blencowe NS Boutron I . RoB 2: a revised tool for assessing risk of bias in randomised trials. BMJ (Clin Res Ed). (2019) 366:l4898. doi: 10.1136/bmj.l4898 31462531

[23] Liu X Deliu N Chakraborty B. Microrandomized trials: developing Just-in-Time Adaptive Interventions for better public health. Am J Public Health. (2023) 113:60–9. doi: 10.2105/AJPH.2022.307150, 36413704 PMC9755932

[24] Sterne JA Hernán MA Reeves BC Savović J Berkman ND Viswanathan M . ROBINS-I: a tool for assessing risk of bias in non-randomised studies of interventions. BMJ (Clin Res Ed). (2016) 355:i4919. doi: 10.1136/bmj.i4919 PMC5062054 27733354

[25] Haag D. Kumar D. Gruber S. Hofer D. P. Sareban M. Treff G. . The last JITAI? Exploring large language models for issuing Just-in-Time Adaptive Interventions: fostering physical activity in a prospective cardiac rehabilitation setting. Proceedings of the 2025 CHI Conference on Human Factors in Computing Systems. (2025). New York, NY, USA: Association for Computing Machinery (ACM).

[26] Sanal-Hayes NE Hayes LD Mair JL Iacono AD Ingram J Mclaughlin M . A digital platform with activity tracking for energy management support in long COVID: a randomised controlled trial. Nat Commun. (2026) 17:945. doi: 10.1038/s41467-025-64831-y PMC12864992 41629267

[27] Hedderson MM Brown SD Quesenberry CP Xu F Liu EF Li KL . Adaptive mobile health intervention to reduce excess gestational weight gain. JAMA Netw Open. (2026) 9:e2680074. doi: 10.1001/jamanetworkopen.2026.8007 PMC13096985 42008264

[28] Wu Y Ma Y Zhang C Wang C Zhang S Zhao M . Effectiveness of a digital technology-assisted personalized exercise prescription in the telerehabilitation of postoperative coronary heart disease patients: a randomized controlled trial. Int J Nurs Sci. (2026) 13:11–8. doi: 10.1016/j.ijnss.2025.12.010 PMC12891784 41684613

[29] Golbus JR Dorsch MP Chen Y Basu T Luff E Klasnja P . Impact of push notifications on physical activity and sodium intake among patients with hypertension: microrandomized trial of a Just-in-Time Adaptive Intervention. J Med Internet Res. (2026) 28:e78218. doi: 10.2196/78218 PMC12779098 41499691

[30] Vos AL de Bruijn GJ Klein M Boerman SC Stuber JM Smit EG. Effectiveness of a just-in-time adaptive app to increase daily steps: an RCT. Am J Prev Med. (2025) 68:154–63. doi: 10.1016/j.amepre.2024.09.010 39299494

[31] Seiterö A Henriksson P Thomas K Henriksson H Löf M Bendtsen M . Effectiveness of a mobile phone-delivered multiple health behavior change intervention (LIFE4YOUth) in adolescents: randomized controlled trial. J Med Internet Res. (2025) 27:e69425. doi: 10.2196/69425 PMC12056421 40262133

[32] Oba T Moriishi C Takano K Katahira K Kimura K. Mobile-based ecological momentary intervention for improving physical activity in adults without regular physical activity: pilot randomized controlled trial. JMIR Form Res. (2025) 9:e79360. doi: 10.2196/79360 PMC12677381 41343702

[33] Dorsch MP Golbus JR Stevens R Trumpower B Basu T Luff E . Physical activity and diet Just-in-Time Adaptive Intervention to reduce blood pressure: a randomized controlled trial. NPJ Digit Med. (2025) 8:438. doi: 10.1038/s41746-025-01844-3 PMC12260109 40659778

[34] Ikegaya M Foo JC Murata T Oshima K Kim J. Using personalized intervention criteria in a mobile Just-in-Time Adaptive Intervention for increasing physical activity in university students: pilot study. JMIR Hum Factors. (2025) 12:e66750. doi: 10.2196/66750 PMC12129369 40418819

[35] Celano CM Healy BC Jacobson LH Bell M Carrillo A Massey CN . An adaptive text message intervention to promote psychological well-being and reduce cardiac risk: the Text4Health controlled clinical pilot trial. J Psychosom Res. (2024) 177:111583. doi: 10.1016/j.jpsychores.2023.111583 PMC12035677 38171212

[36] Bronas UG Marquez DX Fritschi C Petrarca K Kitsiou S Ajilore O . Ecological momentary intervention to replace sedentary time with physical activity to improve executive function in midlife and older Latino adults: pilot randomized controlled trial. J Med Internet Res. (2024) 26:e55079. doi: 10.2196/55079 PMC11413544 39235836

[37] Coppens I De Pessemier T Martens L. Exploring the added effect of three recommender system techniques in mobile health interventions for physical activity: a longitudinal randomized controlled trial. User Model User-Adapt Interact. (2024) 34:1835–90. doi: 10.1007/s11257-024-09407-z

[38] Hendrickx W Wondergem R Veenhof C English C Visser-Meily JMA Pisters MF. Improving movement behavior in people after stroke with the RISE intervention: a randomized multiple baseline study. J Clin Med. (2024) 13:4341. doi: 10.3390/jcm13154341 PMC11313465 39124608

[39] Wafi AM Zaeri MA Khudier AA Abushara AN Adawi MM Zakri LA . Real-time vibration feedback from a smartphone application reduces sedentary time but does not increase physical activity among medical students. Healthcare Basel. (2024) 12:2133. doi: 10.3390/healthcare12212133 PMC11545671 39517345

[40] Golbus JR Shi J Gupta K Stevens R Jeganathan VSE Luff E . Text messages to promote physical activity in patients with cardiovascular disease: a micro-randomized trial of a Just-in-Time Adaptive Intervention. Circ Cardiovasc Qual Outcomes. (2024) 17:e010731. doi: 10.1161/CIRCOUTCOMES.123.010731 PMC11251861 38887953

[41] Fiedler J Seiferth C Eckert T Woll A Wunsch K. A Just-in-Time Adaptive Intervention to enhance physical activity in the SMARTFAMILY2.0 trial. Sport Exerc Perform Psychol. (2023) 12:43–57. doi: 10.1037/spy0000311

[42] Golbus JR Gupta K Stevens R Jeganathan VSE Luff E Shi J . A randomized trial of a mobile health intervention to augment cardiac rehabilitation. NPJ Digit Med. (2023) 6:173. doi: 10.1038/s41746-023-00921-9 PMC10502072 37709933

[43] Valle CG Diamond MA Heiling HM Deal AM Hales DP Nezami BT . Effect of an mHealth intervention on physical activity outcomes among young adult cancer survivors: the IMPACT randomized controlled trial. Cancer-Am Cancer Soc. (2023) 129:461–72. doi: 10.1002/cncr.34556 PMC9834757 36444676

[44] Mair JL Hayes LD Campbell AK Buchan DS Easton C Sculthorpe N. A personalized smartphone-delivered Just-in-Time Adaptive Intervention (JitaBug) to increase physical activity in older adults: mixed methods feasibility study. JMIR Form Res. (2022) 6:e34662. doi: 10.2196/34662 PMC9030994 35389348

[45] Thomas T Xu F Sridhar S Sedgwick T Nkemere L Badon SE . A web-based mHealth intervention with telephone support to increase physical activity among pregnant patients with overweight or obesity: feasibility randomized controlled trial. JMIR Form Res. (2022) 6:e33929. doi: 10.2196/33929 PMC9260523 35731565

[46] Ismail T Al Thani D. Design and evaluation of a Just-in-Time Adaptive Intervention (JITAI) to reduce sedentary behavior at work: experimental study. JMIR Form Res. (2022) 6:e34309. doi: 10.2196/34309 PMC8943689 35080498

[47] Wang J. Fang Y. Frank E. Walton M. A. Burmeister M. Tewari A. . Effectiveness of Gamified Team Competition in the Context of mHealth Intervention for Medical Interns: A Micro-Randomized Trial. Berlin/Heidelberg, Germany: Springer Nature. 4.(2022). 10.1038/s41746-022-00746-y PMC9834206 36631665

[48] Sporrel K Wang S Ettema DDF Nibbeling N Krose BJA Deutekom M . Just-in-time prompts for running, walking, and performing strength exercises in the built environment: 4-week randomized feasibility study. JMIR Form Res. (2022) 6:e35268. doi: 10.2196/35268 PMC9379785 35916693

[49] Klasnja P Rosenberg DE Zhou J Anau J Gupta A Arterburn DE. A quality-improvement optimization pilot of BariFit, a mobile health intervention to promote physical activity after bariatric surgery. Transl Behav Med. (2021) 11:530–9. doi: 10.1093/tbm/ibaa040 32421187

[50] Cushing CC Bejarano CM Ortega A Sayre N Fedele DA Smyth JM. Adaptive mHealth intervention for adolescent physical activity promotion. J Pediatr Psychol. (2021) 46:536–46. doi: 10.1093/jpepsy/jsaa125 33484137

[51] Direito A Tooley M Hinbarji M Albatal R Jiang Y Whittaker R . Tailored daily activity: an adaptive physical activity smartphone intervention. Telemed E-Health. (2020) 26:426–37. doi: 10.1089/tmj.2019.0034 31063038

[52] Klasnja P Smith S Seewald NJ Lee A Hall K Luers B . Efficacy of contextually tailored suggestions for physical activity: a micro-randomized optimization trial of HeartSteps. Ann Behav Med. (2019) 53:573–82. doi: 10.1093/abm/kay067 PMC6401341 30192907

[53] Hiremath SV Amiri AM Thapa-Chhetry B Snethen G Schmidt-Read M Ramos-Lamboy M . Mobile health-based physical activity intervention for individuals with spinal cord injury in the community: a pilot study. PLoS One. (2019) 14:e0223762. doi: 10.1371/journal.pone.0223762 PMC6793862 31613909

[54] Gabrys L Sperzel S Bernhoerster M Banzer W Vogt L. Real-time visual activity feedback for physical activity improvement in breast and colon cancer patients. Res Sports Med. (2017) 25:1–10. doi: 10.1080/15438627.2016.1258639 27882777

[55] Lim S Kang SM Kim KM Moon JH Choi SH Hwang H . Multifactorial intervention in diabetes care using real-time monitoring and tailored feedback in type 2 diabetes. Acta Diabetol. (2016) 53:189–98. doi: 10.1007/s00592-015-0754-8 25936739

[56] Tabak M Op Den Akker H Hermens H. Motivational cues as real-time feedback for changing daily activity behavior of patients with COPD. Patient Educ Couns. (2014) 94:372–8. doi: 10.1016/j.pec.2013.10.014 24332934

[57] King AC Hekler EB Grieco LA Winter SJ Sheats JL Buman MP . Harnessing different motivational frames via mobile phones to promote daily physical activity and reduce sedentary behavior in aging adults. PLoS One. (2013) 8:e62613. doi: 10.1371/journal.pone.0062613 PMC3636222 23638127

[58] Xu Y Liao M Zhang M. Effects of digital health interventions on objectively measured physical activity during the perinatal period: a systematic review and meta-analysis. Front Public Health. (2026) 14:1786474. doi: 10.3389/fpubh.2026.1786474, 42007337 PMC13083171

