# Supplementary material for: Just-in-Time Adaptive Interventions for physical activity: a systematic review of their public health impact and methodological quality
Source: Front Public Health. 2026 Sep 4;14:1934024. doi: 10.3389/fpubh.2026.1934024 (PMC13585906; doi:10.3389/fpubh.2026.1934024)
Supplement: Supplementary file 1 [file Data_Sheet_1.DOCX]

**PubMed：**

| Search number | Query | Sort By | Filters | Search Details | Results | Time | Date |
| --- | --- | --- | --- | --- | --- | --- | --- |
| 5 | (("Exercise"[Mesh]) OR (Physical Activity [tw] OR Exercise [tw] OR Physical fit*[tw] OR Sport* participation[tw] OR Sport* activit* [tw] OR Active lifestyle[tw] OR Movement behavio*[tw] OR MVPA[tw] OR Moderate to vigorous physical activity [tw] OR Step count* [tw] OR Energy expenditur*[tw] OR Daily activit* [tw] OR Walk* [tw] OR Jog[tw] OR Run)) AND ("just-in-time"[Title/Abstract] OR "JITAI*"[Title/Abstract] OR "ecological momentary intervention*"[Title/Abstract] OR "context-aware*"[Title/Abstract] OR "adaptive intervention"[Title/Abstract:~4] OR "adaptive interventions"[Title/Abstract:~4] OR "digital behavior change"[Title/Abstract] OR "digital behaviour change"[Title/Abstract] OR "DBCI*"[Title/Abstract] OR "dynamic tailoring"[Title/Abstract:~4] OR "dynamically tailored"[Title/Abstract:~4] OR "real-time tailoring"[Title/Abstract:~4] OR "real-time therap*"[Title/Abstract] OR "tailored feedback"[Title/Abstract:~4] OR "adaptive feedback"[Title/Abstract:~4] OR "real-time feedback"[Title/Abstract:~4] OR "microrandomi*"[Title/Abstract] OR "micro-randomi*"[Title/Abstract]) |  |  | ("Exercise"[MeSH Terms] OR ("physical activity"[Text Word] OR "Exercise"[Text Word] OR "physical fit*"[Text Word] OR "sport* participation"[Text Word] OR "sport* activit*"[Text Word] OR "active lifestyle"[Text Word] OR "movement behavio*"[Text Word] OR "MVPA"[Text Word] OR "moderate to vigorous physical activity"[Text Word] OR "step count*"[Text Word] OR "energy expenditur*"[Text Word] OR "daily activit*"[Text Word] OR "walk*"[Text Word] OR "Jog"[Text Word] OR "Run"[All Fields])) AND ("just-in-time"[Title/Abstract] OR "jitai*"[Title/Abstract] OR "ecological momentary intervention*"[Title/Abstract] OR "context aware*"[Title/Abstract] OR "adaptive intervention"[Title/Abstract:~4] OR "adaptive interventions"[Title/Abstract:~4] OR "digital behavior change"[Title/Abstract] OR "digital behaviour change"[Title/Abstract] OR "dbci*"[Title/Abstract] OR "dynamic tailoring"[Title/Abstract:~4] OR "dynamically tailored"[Title/Abstract:~4] OR "real-time tailoring"[Title/Abstract:~4] OR "real time therap*"[Title/Abstract] OR "tailored feedback"[Title/Abstract:~4] OR "adaptive feedback"[Title/Abstract:~4] OR "real-time feedback"[Title/Abstract:~4] OR "microrandomi*"[Title/Abstract] OR "micro randomi*"[Title/Abstract]) | 1,658 | 2:50:18 | 2026/6/1 |
| 4 | "just-in-time"[Title/Abstract] OR "JITAI*"[Title/Abstract] OR "ecological momentary intervention*"[Title/Abstract] OR "context-aware*"[Title/Abstract] OR "adaptive intervention"[Title/Abstract:~4] OR "adaptive interventions"[Title/Abstract:~4] OR "digital behavior change"[Title/Abstract] OR "digital behaviour change"[Title/Abstract] OR "DBCI*"[Title/Abstract] OR "dynamic tailoring"[Title/Abstract:~4] OR "dynamically tailored"[Title/Abstract:~4] OR "real-time tailoring"[Title/Abstract:~4] OR "real-time therap*"[Title/Abstract] OR "tailored feedback"[Title/Abstract:~4] OR "adaptive feedback"[Title/Abstract:~4] OR "real-time feedback"[Title/Abstract:~4] OR "microrandomi*"[Title/Abstract] OR "micro-randomi*"[Title/Abstract] |  |  | "just-in-time"[Title/Abstract] OR "jitai*"[Title/Abstract] OR "ecological momentary intervention*"[Title/Abstract] OR "context aware*"[Title/Abstract] OR "adaptive intervention"[Title/Abstract:~4] OR "adaptive interventions"[Title/Abstract:~4] OR "digital behavior change"[Title/Abstract] OR "digital behaviour change"[Title/Abstract] OR "dbci*"[Title/Abstract] OR "dynamic tailoring"[Title/Abstract:~4] OR "dynamically tailored"[Title/Abstract:~4] OR "real-time tailoring"[Title/Abstract:~4] OR "real time therap*"[Title/Abstract] OR "tailored feedback"[Title/Abstract:~4] OR "adaptive feedback"[Title/Abstract:~4] OR "real-time feedback"[Title/Abstract:~4] OR "microrandomi*"[Title/Abstract] OR "micro randomi*"[Title/Abstract] | 14,323 | 2:28:11 | 2026/6/1 |
| 3 | ("Exercise"[Mesh]) OR (Physical Activity [tw] OR Exercise [tw] OR Physical fit*[tw] OR Sport* participation[tw] OR Sport* activit* [tw] OR Active lifestyle[tw] OR Movement behavio*[tw] OR MVPA[tw] OR Moderate to vigorous physical activity [tw] OR Step count* [tw] OR Energy expenditur*[tw] OR Daily activit* [tw] OR Walk* [tw] OR Jog[tw] OR Run) |  |  | "Exercise"[MeSH Terms] OR "physical activity"[Text Word] OR "Exercise"[Text Word] OR "physical fit*"[Text Word] OR "sport* participation"[Text Word] OR "sport* activit*"[Text Word] OR "active lifestyle"[Text Word] OR "movement behavio*"[Text Word] OR "MVPA"[Text Word] OR "moderate to vigorous physical activity"[Text Word] OR "step count*"[Text Word] OR "energy expenditur*"[Text Word] OR "daily activit*"[Text Word] OR "walk*"[Text Word] OR "Jog"[Text Word] OR "Run"[All Fields] | 968,470 | 2:27:00 | 2026/6/1 |
| 2 | Physical Activity [tw] OR Exercise [tw] OR Physical fit*[tw] OR Sport* participation[tw] OR Sport* activit* [tw] OR Active lifestyle[tw] OR Movement behavio*[tw] OR MVPA[tw] OR Moderate to vigorous physical activity [tw] OR Step count* [tw] OR Energy expenditur*[tw] OR Daily activit* [tw] OR Walk* [tw] OR Jog[tw] OR Run |  |  | "physical activity"[Text Word] OR "Exercise"[Text Word] OR "physical fit*"[Text Word] OR "sport* participation"[Text Word] OR "sport* activit*"[Text Word] OR "active lifestyle"[Text Word] OR "movement behavio*"[Text Word] OR "MVPA"[Text Word] OR "moderate to vigorous physical activity"[Text Word] OR "step count*"[Text Word] OR "energy expenditur*"[Text Word] OR "daily activit*"[Text Word] OR "walk*"[Text Word] OR "Jog"[Text Word] OR "Run"[All Fields] | 931,417 | 2:26:48 | 2026/6/1 |
| 1 | "Exercise"[Mesh] |  |  | "Exercise"[MeSH Terms] | 287,284 | 2:25:34 | 2026/6/1 |

**Web of Science**

| Entitlements | # | Search Query | Database | Results | Date Run |
| --- | --- | --- | --- | --- | --- |
| - WOS.IC: 1993 to 2026 - WOS.CCR: 1985 to 2026 - WOS.SCI: 2003 to 2026 | 6 | TS=("Exercise" OR "Physical Activity" OR "Physical fit*" OR "Sport* participation" OR "Sport* activit*" OR "Active lifestyle" OR "Movement behavio*" OR "MVPA" OR "Moderate to vigorous physical activity" OR "Step count*" OR "Energy expenditur*" OR "Daily activit*" OR "Walk*" OR "Jog" OR "Run") | Web of Science Core Collection | 942888 | Mon Jun 01 2026 18:47:26 GMT+0800 |
| - WOS.IC: 1993 to 2026 - WOS.CCR: 1985 to 2026 - WOS.SCI: 2003 to 2026 | 7 | TI=("just-in-time" OR “jitai*” OR "ecological momentary intervention*" OR “context-aware*” OR “adaptive NEAR/4 intervention*“ OR "digital behavio*r change" OR dbci* OR “dynamic* NEAR/4 tailor*” OR “real-time NEAR/4 tailoring” OR "real-time therap*" OR “tailor* NEAR/4 feedback” OR “adapt* NEAR/4 feedback“ OR “real-time NEAR/4 feedback” OR “microrandomi*” OR “micro-randomi*”) OR AB=("just-in-time" OR jitai* OR "ecological momentary intervention*" OR context-aware* OR ( adaptive NEAR/4 intervention* ) OR "digital behavio*r change" OR dbci* OR “dynamic* NEAR/4 tailor*” OR “real-time NEAR/4 tailoring” OR "real-time therap*" OR “tailor* NEAR/4 feedback” OR “adapt* NEAR/4 feedback” OR “real-time NEAR/4 feedback” OR microrandomi* OR micro-randomi*) | Web of Science Core Collection | 14685 | Mon Jun 01 2026 18:47:47 GMT+0800 |
| - WOS.IC: 1993 to 2026 - WOS.CCR: 1985 to 2026 - WOS.SCI: 2003 to 2026 | 8 | #6 AND #7 | Web of Science Core Collection | 859 | Mon Jun 01 2026 18:47:57 GMT+0800 |

**Embase:**

'exercise'/exp OR 'Physical Activity':ti,ab,kw OR 'Physical fit*':ti,ab,kw OR 'Sport* participation':ti,ab,kw OR 'Sport* activit*':ti,ab,kw OR 'Active lifestyle':ti,ab,kw OR 'Movement behavio*':ti,ab,kw OR 'MVPA':ti,ab,kw OR 'Moderate to vigorous physical activity':ti,ab,kw OR 'Step count*':ti,ab,kw OR 'Energy expenditur*':ti,ab,kw OR 'Daily activit*':ti,ab,kw OR 'Walk*':ti,ab,kw OR 'Jog':ti,ab,kw OR 'Run':ti,ab,kw

'just-in-time':ab,ti OR 'jitai$':ab,ti OR 'ecological momentary intervention$':ab,ti OR 'context-aware*':ab,ti OR ((adaptive NEAR/5 intervention$):ab,ti) OR 'digital behavio$r change':ab,ti OR 'dbci$':ab,ti OR ((dynamic* NEAR/5 tailor*):ab,ti) OR (('real time' NEAR/4 tailoring):ab,ti) OR 'real-time therap*':ab,ti OR ((tailor* NEAR/5 feedback):ab,ti) OR ((adapt* NEAR/5 feedback):ab,ti) OR (('real time' NEAR/5 feedback):ab,ti) OR 'microrandomi*':ab,ti OR 'micro-randomi*':ab,ti

**Cochrane CENTRAL**

("Exercise"):ti,ab,kw OR ("Physical Activity"):ti,ab,kw OR ("Physical fit*"):ti,ab,kw OR ("Sport* participation"):ti,ab,kw OR ("Sport* activit*"):ti,ab,kw OR ("Active lifestyle"):ti,ab,kw OR ("Movement behavio*"):ti,ab,kw OR ("MVPA"):ti,ab,kw OR ("Moderate to vigorous physical activity"):ti,ab,kw OR ("Step count*"):ti,ab,kw OR ("Energy expenditur*"):ti,ab,kw OR ("Daily activit*"):ti,ab,kw OR ("Walk*"):ti,ab,kw OR ("Jog"):ti,ab,kw OR ("Run"):ti,ab,kw

("just-in-time" OR JITAI? OR (ecological NEXT momentary NEXT intervention?) OR context-aware* OR (adaptive NEAR/5 intervention?) OR (digital NEXT behavio?r NEXT change) OR dbci? OR (dynamic* NEAR/5 tailor*) OR (real-time NEAR/5 tailoring) OR (real-time NEXT therap*) OR (tailor* NEAR/5 feedback) OR (adapt* NEAR/5 feedback) OR (real-time NEAR/5 feedback) OR microrandomi* OR micro-randomi*):ti,ab,kw

**CINAHL**

TI("Exercise" OR "Physical Activity" OR "Physical fit*" OR "Sport* participation" OR "Sport* activit*" OR "Active lifestyle" OR "Movement behavio*" OR "MVPA" OR "Moderate to vigorous physical activity" OR "Step count*" OR "Energy expenditur*" OR "Daily activit*" OR "Walk*" OR "Jog" OR "Run") AND (TI("just-in-time" OR jitai* OR "ecological momentary" OR context-aware* OR "adaptive intervention" OR "digital behavio*r change" OR dbci* OR "dynamic* tailor*" OR "real-time tailoring" OR "real-time therap*" OR "tailor* feedback" OR "adapt* feedback" OR "real-time feedback" OR microrandomi* OR micro-randomi*) OR AB("just-in-time" OR jitai* OR "ecological momentary" OR context-aware* OR "adaptive intervention" OR "digital behavio*r change" OR dbci* OR "dynamic* tailor*" OR "real-time tailoring" OR "real-time therap*" OR "tailor* feedback" OR "adapt* feedback" OR "real-time feedback" OR microrandomi* OR micro-randomi*))

**PsycINFO**

TI("Exercise" OR "Physical Activity" OR "Physical fit*" OR "Sport* participation" OR "Sport* activit*" OR "Active lifestyle" OR "Movement behavio*" OR "MVPA" OR "Moderate to vigorous physical activity" OR "Step count*" OR "Energy expenditur*" OR "Daily activit*" OR "Walk*" OR "Jog" OR "Run") AND (TI("just-in-time" OR jitai* OR "ecological momentary" OR context-aware* OR "adaptive intervention" OR "digital behavio*r change" OR dbci* OR "dynamic* tailor*" OR "real-time tailoring" OR "real-time therap*" OR "tailor* feedback" OR "adapt* feedback" OR "real-time feedback" OR microrandomi* OR micro-randomi*) OR AB("just-in-time" OR jitai* OR "ecological momentary" OR context-aware* OR "adaptive intervention" OR "digital behavio*r change" OR dbci* OR "dynamic* tailor*" OR "real-time tailoring" OR "real-time therap*" OR "tailor* feedback" OR "adapt* feedback" OR "real-time feedback" OR microrandomi* OR micro-randomi*))

**Scopus:**

TITLE ( "Exercise" OR "Physical Activity" OR "Physical fit*" OR "Sport* participation" OR "Sport* activit*" OR "Active lifestyle" OR "Movement behavio*" OR "MVPA" OR "Moderate to vigorous physical activity" OR "Step count*" OR "Energy expenditur*" OR "Daily activit*" OR "Walk*" OR "Jog" OR "Run" ) AND TITLE-ABS ( "just-in-time" OR jitai* OR "ecological momentary" OR context-aware* OR ( adaptive W/4 intervention* ) OR "digital behavio*r change" OR dbci* OR ( dynamic* W/4 tailor* ) OR ( real-time W/4 tailoring ) OR "real-time therap*" OR ( tailor* W/4 feedback ) OR ( adapt* W/4 feedback ) OR ( real-time W/4 feedback ) OR microrandomi* OR micro-randomi* )

Supplementary searches

| Database | Search string |
| --- | --- |
| **ACM Digital Library** | ("just-in-time adaptive intervention" OR JITAI) AND (Title:"physical activity" OR Title:exercise OR Title:walk* OR Title:step OR Title:MVPA OR Abstract:"physical activity" OR Abstract:exercise OR Abstract:walk* OR Abstract:step OR Abstract:MVPA) |
| **DBLP** | "just in time adaptive intervention" (39 matches) + JITAI (38 matches) — searched separately due to platform limitations |
| [**ClinicalTrials.gov**](https://clinicaltrials.gov/) | AREA[InterventionSearch]("just-in-time adaptive intervention" OR JITAI OR "ecological momentary intervention" OR "context-aware" OR "adaptive intervention") AND AREA[OutcomeSearch]("physical activity" OR exercise OR steps OR MVPA) |
| **ISRCTN** | "just-in-time adaptive intervention" OR JITAI |
